# Effect of Different Anticoagulant Agents on Immune-Related Genes in Leukocytes Isolated from the Whole-Blood of Holstein Cows

**DOI:** 10.3390/genes14020406

**Published:** 2023-02-04

**Authors:** Viviana Floridia, Marta Sfulcini, Enrico D’Alessandro, Luca Cattaneo, Matteo Mezzetti, Luigi Liotta, Erminio Trevisi, Vincenzo Lopreiato, Andrea Minuti

**Affiliations:** 1 Department of Veterinary Sciences, Università di Messina, Viale Palatucci, 13, 98168 Messina, Italy; 2 Department of Animal Sciences, Food and Nutrition, Faculty of Agriculture, Food and Environmental Science, Università Cattolica del Sacro Cuore, 29122 Piacenza, Italy

**Keywords:** Holstein, leukocytes, EDTA, heparin, Na-citrate, gene expression

## Abstract

Anticoagulants, such as ethylenediaminetetraacetic acid (EDTA), sodium citrate (Na-citrate), or heparin are normally used in hematological clinical tests to prevent coagulation. Although anticoagulants are fundamental for the correct application of clinical tests, they produce adverse effects in different fields, such as those involving specific molecular techniques; for instance, quantitative real time polymerase chain reactions (qPCR) and gene expression evaluation. For this reason, the aim of this study was to evaluate the expression of 14 genes in leukocytes that were isolated from the blood of Holstein cows, and which were collected in Li-heparin, K-EDTA, or Na-citrate tubes; then, they were analyzed using qPCR. Only the *SDHA* gene showed a significant dependence (*p* ≤ 0.05) on the anticoagulant that was used with the lowest expression; this was observed in Na-Citrate after being compared with Li-heparin and K-EDTA (*p* < 0.05). Although a variation in transcript abundance with the three anticoagulants was observed in almost all the investigated genes, the relative abundance levels were not statistically significant. In conclusion, the qPCR results were not influenced by the presence of the anticoagulant; thus, we had the opportunity to choose the test tube that was used in the experiment without interfering effects impacting the gene expression levels caused by the anticoagulant.

## 1. Introduction

Blood is one of the most commonly used samples for research, and its collection is considered to be minimally invasive when using the standard procedures currently available. Anticoagulants are chemical substances that are normally used in haematological clinical tests because they preserve blood and prevent coagulation [1]. The most widely used anticoagulants are ethylenediamintetraceetic acid (EDTA), sodium citrate (Na-citrate), and heparin; they work by binding calcium ions (EDTA and citrate) or by inhibiting thrombin (heparin) [2]. Unfortunately, they could negatively affect some assessments [1,2,3,4] such as biochemical blood values [2,5], concentrations of different blood cations [3,6], and/or blood cell counts [7,8]. Several studies have reported that the use of anticoagulants can also influence the measurement of transcripts [9,10,11,12]. For example, unprocessed EDTA blood samples that have been stored for a long time can cause alterations in the levels of the interleukin-1 receptor antagonist (IL-1ra) [11]. Moreover, Sefrioui et al. [13] reported that the addition of heparinase to blood samples overcome the inhibition of the polymerase chain reaction (PCR) process that is caused by heparin.

Furthermore, the National Committee for Research Ethics in Science and Technology (NENT) provides ethical guidelines for researchers with “the three Rs”: (1) replace, (2) reduce, and (3) refine. The guidelines indicate that researchers must minimize the risk of animal suffering and provide high animal welfare standards [14]. Consequently, according to the ethical guidelines, it is essential to minimise the number of blood samples harvested. For this reason, using blood from the same tube for different analyses, instead of taking multiple samples, is still under investigation.

Lithium-heparin (Li-heparin) has always been considered the best type of anticoagulant for obtaining the plasma that is used for clinical chemistry determinations. Real-time polymerase chain reaction (qPCR) is a widely used method, and it is applied in many fields, including gene expression, and it provides precise results [15,16,17,18,19,20]. qPCR is the most sensitive method for studying gene expression. It is widely used in medicine and other scientific fields. With the development of semiquantitative and quantitative methods, this method has been increasingly applied when studying the gene expression of immune- and metabolic-related mediators. An inhibitory effect caused by heparin has been reported; this effect is caused by its interaction with DNA and DNA polymerases. Although both DNA and heparin are highly negatively charged and would not be expected to interact, the binding between these two molecules could be mediated by divalent cations, such as magnesium ions [2].

Unfortunately, there are a lack of studies on the effects of anticoagulants on transcript abundance in cattle. On the other hand, although several investigations were carried out in order to understand the effects of anticoagulants on the amplification of DNA with PCR, no studies have aimed at evaluating the anticoagulant’s effects on cDNA synthesis and cDNA amplification using RNA extraction as a starting point; therefore, the aim of this study was to evaluate the expression of different genes in leukocytes that have been isolated from the blood of Holstein cows, which were collected in K-EDTA, Li-heparin, or Na-citrate tubes, and analysed using the qPCR technique.

## 2. Materials and Methods

The blood samples were collected immediately after the cows’ morning meal, from four mid-lactating Holstein cows via jugular venipuncture. Tubes (BD Vacutainer; BD and Co.) containing the three different anticoagulants, (1) K-EDTA, (2) Na-citrate, and (3) Li-heparin, were used. Cows did not show any clinical diseases, and they were subjected to the same housing and feeding conditions at the Università Cattolica del Sacro Cuore research dairy barn (San Bonico, Piacenza, Italy). Immediately after collection, the tubes were cooled in an ice-water bath. Within 30 min of collection, they were moved to the laboratory to start the RNA extraction procedure. Circulating leukocytes were isolated by washing samples with a red blood cell lysis buffer until a clean white blood cell pellet was obtained; then, 1 mL of Trizol (Invitrogen Corp., CA, USA) was added. The total RNA was then extracted using the RNeasy Mini Kit (Qiagen, Hilden, Germany), and residual DNA was removed using the RNase-Free DNase Set (Qiagen, Hilden, Germany), in accordance with the manufacturer’s protocols.

After extraction, RNA was quantified using the Qubit RNA BR Assay Kit (Thermo Fisher Scientific, Waltham, MA, USA), and RNA quality was assessed using the Experion Automated Electrophoresis System (Bio-Rad, Hercules, CA, USA). The average of the RNA quality index for the 12 samples was 9.8 (range: 9.6–9.8).

Synthesis of cDNA was performed using a reverse transcription kit (RevertAid RT Reverse Transcription Kit; Thermo Fisher Scientific), as previously described [18]. Briefly, each reaction mixture (1 μL of 100 ng/μL of total RNA solution, 1 μL of random hexamer primer, and 9.5 μL of DNase/RNase-free water) was incubated for 5 min at 65 °C. Then, 8.5 μL of master mix (5 μL of 5 × First-Strand Buffer, 1 μL of 0.1 M DTT, 0.25 μL of RevertAid RT (200 U/μL), and 0.25 μL of RiboLock RNase Inhibitor (20 U/μL)) was added. The reaction was performed in an Eppendorf Mastercycler gradient (Westbury, NY), in accordance with the following procedure: 25 °C for 5 min, 42 °C for 60 min, and 70 °C for 5 min. The resultant cDNA was diluted (1:4 (vol/vol) with DNase/RNasefree water), and then stored before quantitative PCR testing. The qPCR procedure was performed using 4 μL of diluted cDNA combined with 6 μL of a mixture that was composed of: 5 μL 1 × SYBR Green Master Mix (Applied Biosystems, Woolston Warrington, UK); 0.4 μL of 10 μM forward and reverse primers, respectively; and 0.2 μL of nuclease-free water. The qPCR reaction was performed in an Optical 384-Well Reaction Plate (CFX384 Touch; Bio-Rad, Hercules, CA, USA) using a specific temperature program: (1) 2 min at 50 °C, (2) 10 min at 95 °C, (3) 40 cycles with 15 s at 95 °C, and (4) 1 min at 60 °C. A dissociation curve was evaluated (gradient from 95 °C to 60 °C to 95 °C) to check the amplicon quality. The qPCR efficiency and quantification cycle values were obtained for each reaction using LinRegPCR (Version 2017.1; Amsterdam UMC, Amsterdam, the Netherlands [21,22]), which is a program for the analysis of quantitative qPCR data based on measurements from SYBR green and similar fluorescent dyes. This program measures and subtracts the baseline fluorescence; then, it provides a window-of-linearity for the calculation of PCR efficiency. Based on the mean PCR efficiency per amplicon, the quantitation cycle (Cq) value per sample, the fluorescence threshold (used to determine the Cq), and the starting concentration per sample (in arbitrary fluorescence units) were calculated [21,22]. The analyzed genes included four internal control genes (*ACTB*, *SDHA*, *YWHAZ*, and *GAPDH*) and genes that are involved in recognition and immune mediation (*TLR2*), cell migration and adhesion (*TLN1*, *ITGB2*, and *LGALS8*), the inflammation process (*IL1B*, *IL1R*, *S100A8*, and *TNF*), and antimicrobial strategies (*LCN2* and *LTF*). Additional details regarding primer information, primer sequencing results, and qPCR amplification efficiency, are included in Supplementary Tables S1–S3.

Arbitrary mRNA abundance data were analyzed using the MIXED procedure of SAS version 9.4 (SAS Institute Inc., Cary, NC, USA), in accordance with the following model:

Yi = μ + Bi + cm:i + εi;

where Yi = dependent continuous variable, μ = overall mean, Bi = fixed effect of anticoagulant (i = K-EDTA vs. Na-citrate vs. Li-heparin), cm = random effect of the *m*^th^ animal (cow), and εi = residual error.

All means were compared using the PDIFF statement of SAS. Significant differences were declared at *p* ≤ 0.05.

## 3. Results

The mRNA abundance of genes investigated in the whole blood leukocytes is shown in Table 1 and Figure S1 of the Supplementary file. Only the *SDHA* gene showed a significant difference (*p* ≤ 0.05). Indeed, its lowest expression was observed in Na-Citrate when compared with Li-heparin and K-EDTA (*p* < 0.05) samples. The average amplification efficiency of the genes under investigation was 1.82 (Supplementary Table S3), regardless type of anticoagulants. The amplification efficiency was, on average, 1.81 (ranged from 1.71 to 1.86) for samples that were collected with K-EDTA, 1.81 (ranged from 1.72 to 1.87) for samples that were collected with Li-Heparin, and 1.82 (ranged from 1.73 to 1.89) for samples that were collected with Na-Citrate.

## 4. Discussion

In this study, we evaluated the effect of three different anticoagulants (Li-Heparin, K-EDTA, and Na-Citrate) on the transcript abundance of 14 genes in the circulating leukocytes of mid-lactating Holstein cows that were analysed using real-time qPCR. These genes code for proteins that are involved in several functions, such as recognition and immune mediation, cell migration and adhesion, inflammation processes, and antimicrobial strategies. In addition, genes used as control genes were also assessed. We did not find any adverse effect on gene expression when different types of anticoagulants were used, a result that is contrary to what has been reported in the literature. For instance, Yokota et al. [23] reported that that most heparin was extracted with DNA that was obtained from white cells, and thus, much care should be taken in PCR assays if the white cell count in the blood samples is insufficient as a result of the amount of heparin present. We used a strict protocol during the sampling and mRNA extraction processes; in particular, we ensured that low temperatures were maintained throughout the experiment to preserve mRNA integrity [24]. Härtel et al. [25] reported that different protocols (for example, differences in blood sampling technique or cell separation) can cause a non-physiological ex vivo induction of cytokine mRNA expression; hence, it is important to follow the same procedure for all samples so that the samples are not affected in different ways. Yokota et al. [23] discovered that the inhibitory effect of heparin on gene amplification is dose-dependent, and they reported that the maximum permissible amount of heparin is 0.106 U for 50 μL of PCR mixture. We calculated the concentration of heparin in the PCR starting mixture using the concentration contained in the tube. Assuming that all of the heparin binds with the extracted mRNA, and after calculating the effect of subsequent dilutions, we obtained a value that was lower than the maximum permissible amount reported [23]. Other authors reported that a standard phenol/chloroform purification can enable the amplification of whole blood samples taken in lithium heparin solutions [26]. Moreover, as reported in Sefrioui et al. [13], the starting cDNA concentration is important. A lower concentration of cDNA leads to a higher heparin-induced inhibitory effect on cDNA amplification; this is due to the dilution effect.

The ethical guidelines for the use of animals in research recommend that the number of samples harvested must be minimal. To achieve this goal, as Li-heparin tubes are most commonly used for the analysis of clinical biochemistry, and because the gene expression results obtained in this study were not influenced by the presence of this anticoagulant, it was possible to use these tubes to collect a single sample, upon which, both gene expression and clinical biochemistry analyses could be performed.

## 5. Conclusions

As previously reported in the literature, the various anticoagulants, especially heparin, can interfere with qPCR analysis; however, tubes that are normally used for blood collection contain an anticoagulant concentration that may be considered below the threshold; this causes interference. Overall, from the present study, it is evident that using different anticoagulants to collect bovine blood samples does not significantly alter gene expression in circulating blood leukocytes; therefore, when collecting bovine blood samples, it is possible to select the best type of test tube without anticoagulant interference impacting mRNA expression values.

## Figures and Tables

**Table 1:** Arbitrary mRNA abundance of genes in leukocytes that were isolated from the blood of mid-lactating Holstein cows; they were collected in Li-heparin, K-EDTA, or Na-citrate tubes.

Target	Li-Heparin	K-EDTA	Na-Citrate	SEM ^1^	*p*-Value ^2^
Recognition and immune mediation					
*TLR2*	775.2	841.9	720.9	79.8	0.58
Cell migration and adhesion					
*TLN1*	6279.1	6780.5	5879.5	1357.7	0.71
*ITGB2*	3611.2	3522.6	3010.0	476.0	0.64
*LGALS8*	333.4	325.6	274.8	26.9	0.30
Inflammation process					
*IL1B*	2463.9	2798.9	3269.9	814.7	0.63
*IL1R*	266.5	337.5	341.1	111.3	0.68
*S100A8*	12,795.0	12,644.0	12,975.0	1824.8	0.98
*TNF*	306.8	289.9	239.3	36.5	0.43
Antimicrobial strategies					
*LCN2*	62.9	63.7	54.1	11.2	0.80
*LTF*	6.19	10.75	9.19	16.18	0.94
Internal control genes					
*GAPDH*	6045.7	5699.7	4892.1	991.5	0.66
*YWHAZ*	2182.8	2103.6	1821.8	456.4	0.61
*ACTB*	82,234.0	89,663.0	68,228.0	20,132.0	0.56
*SDHA*	727.8 ^a^	735.8 ^a^	575.5 ^b^	42.9	0.05

^1^ Greatest SEM. ^2^ *p*-values for the anticoagulant effect. ^a,b^ Values within a row with different superscripts indicate statistical difference (*p* < 0.05).

## Data Availability

The data that support the findings of this study are available from the corresponding authors upon request.

## Supplementary Materials

The following supporting information can be downloaded at: https://www.mdpi.com/article/10.3390/genes14020406/s1, Table S1: GenBank accession number, sequence, and amplicon size of primers used to analyze gene expression by quantitative PCR; Table S2: Sequencing results obtained from the PCR product, the *Bos taurus* specific primer, for use with genes, is under investigation. Table S3: Average of the amplification efficiencies of the genes under investigation. Figure S1: Arbitrary mRNA abundance of genes in leukocytes that were isolated from the blood of mid-lactating Holstein dairy cow.

## References

[1] NIELSEN H. (1985). Influence of five different anticoagulants on human blood monocyte isolation and functional activities. Acta Pathol. Microbiol. Immunol. Scand. C.

[2] Mohri M., Shakeri H., Lotfollah Zadeh S. (2007). Effects of common anticoagulants (heparin, citrate and EDTA) on routine plasma biochemistry of cattle. Comp. Clin. Path..

[3] Banfi G., Salvagno G.L., Lippi G. (2022). The role of ethylenediamine tetraacetic acid (EDTA) as in vitro anticoagulant for diagnostic purposes. Clin. Chem. Lab. Med..

[4] Goossens W., Van Duppen V., Verwilghen R.L. (1991). K2- or K3-EDTA: The anticoagulant of choice in routine haematology?. Clin. Lab. Haematol..

[5] Luethy D., Hopster K. (2020). Electrolyte measurement in goats: Comparison of 2 blood gas analyzers and evaluation of the preanalytical blood sample preparation on measurement results. Tierarztl. Prax. Ausg. G Grosstiere. Nutztiere..

[6] Higgins C. (2007). The use of heparin in preparing samples for blood-gas analysis. MLO. Med. Lab. Obs..

[7] Stokol T., Erb H.N. (2007). A comparison of platelet parameters in EDTA- and citrate-anticoagulated blood in dogs. Vet. Clin. Pathol..

[8] Raczat T., Kraemer L., Gall C., Weiss D.R., Eckstein R., Ringwald J. (2014). The influence of four different anticoagulants on dynamic light scattering of platelets. Vox Sang..

[9] Shalekoff S., Page-Shipp L., Tiemessen C.T. (1998). Effects of anticoagulants and temperature on expression of activation markers CD11b and HLA-DR on human leukocytes. Clin. Diagn. Lab. Immunol..

[10] Moniuszko M., Kowal K., Rusak M., Pietruczuk M., Dabrowska M., Bodzenta-Lukaszyk A. (2006). Monocyte CD163 and CD36 expression in human whole blood and isolated mononuclear cell samples: Influence of different anticoagulants. Clin. Vaccine Immunol..

[11] Aziz N., Butch A.W., Ryner T.C., Martinez-Maza O., Detels R. (2019). The influence of EDTA Vacutainer blood collection tube on the level of blood interleukin-1 receptor antagonist. J. Immunol. Methods.

[12] Ibeagha-Awemu E.M., Ibeagha A.E., Zhao X. (2012). The influence of different anticoagulants and sample preparation methods on measurement of mCD14 on bovine monocytes and polymorphonuclear neutrophil leukocytes. BMC Res. Notes.

[13] Sefrioui D., Beaussire L., Clatot F., Delacour J., Perdrix A., Frebourg T., Michel P., Di Fiore F., Sarafan-Vasseur N. (2017). Heparinase enables reliable quantification of circulating tumor DNA from heparinized plasma samples by droplet digital PCR. Clin. Chim. Acta.

[14] The National Committee for Research Ethics *Ethical Guidelines for the Use of Animals in Research.* The National Committee for Research Ethics: 2019. https://www.forskningsetikk.no/en/guidelines/science-and-technology/ethical-guidelines-for-the-use-of-animals-in-research/.

[15] Candotti D., Temple J., Owusu-Ofori S., Allain J.P. (2004). Multiplex real-time quantitative RT-PCR assay for hepatitis B virus, hepatitis C virus, and human immunodeficiency virus type 1. J. Virol. Methods.

[16] Green M.R., Sambrook J. (2018). Analysis and Normalization of Real-Time Polymerase Chain Reaction (PCR) Experimental Data. Cold Spring Harb. Protoc..

[17] Galluzzi L., Ceccarelli M., Diotallevi A., Menotta M., Magnani M. (2018). Real-time PCR applications for diagnosis of leishmaniasis. Parasit. Vectors.

[18] Cattaneo L., Mezzetti M., Lopreiato V., Piccioli-Cappelli F., Trevisi E., Minuti A. (2021). Gene network expression of whole blood leukocytes in dairy cows with different milk yield at dry-off. PLoS ONE.

[19] Lopreiato V., Minuti A., Morittu V.M., Britti D., Piccioli-Cappelli F., Loor J.J., Trevisi E. (2020). Short communication: Inflammation, migration, and cell-cell interaction-related gene network expression in leukocytes is enhanced in Simmental compared with Holstein dairy cows after calving. J. Dairy Sci..

[20] Valasek M.A., Repa J.J. (2005). The power of real-time PCR. Am. J. Physiol.-Adv. Physiol. Educ..

[21] Ruijter J.M., Ramakers C., Hoogaars W.M., Karlen Y., Bakker O., van den Hoff M.J., Moorman A.F. (2009). Amplification efficiency: Linking baseline and bias in the analysis of quantitative PCR data. Nucleic. Acids. Res..

[22] Untergasser A., Ruijter J.M., Benes V., van den Hoff M.J.B. (2021). Web-based LinRegPCR: Application for the visualization and analysis of (RT)-qPCR amplification and melting data. BMC Bioinform..

[23] Yokota M., Tatsumi N., Nathalang O., Yamada T., Tsuda I. (1999). Effects of heparin on polymerase chain reaction for blood white cells. J. Clin. Lab. Anal..

[24] Jung R., Lübcke C., Wagener C., Neumaier M. (1997). Reversal of RT-PCR Inhibition Observed in Heparinized Clinical Specimens. Biotechniques.

[25] Härtel C., Bein G., Müller-Steinhardt M., Klüter H. (2001). Ex vivo induction of cytokine mRNA expression in human blood samples. J. Immunol. Methods.

[26] Vaughan-Shaw P.G., Walker M., Ooi L., Gilbert N., Farrington S.M., Dunlop M.G. (2015). A simple method to overcome the inhibitory effect of heparin on DNA amplification. Cell. Oncol..

