# Supplementary material for: Effect of Different Anticoagulant Agents on Immune-Related Genes in Leukocytes Isolated from the Whole-Blood of Holstein Cows
Source: Genes (Basel). 2023 Feb 4;14(2):406. doi: 10.3390/genes14020406 (PMC9957540; doi:10.3390/genes14020406)
Supplement: Supplementary file 1 [file genes-14-00406-s001.zip › genes-2183406-supplementary.pdf]

Supplementary Table S1. GenBank accession number, sequence and amplicon size of primers used to analyze gene expression by quantitative PCR.

Accession no.	Gene	Primers[1]	Primers (5'-3')	Length (bp)[2]
NM_175781.1	*ITGB2*	F.1231	GACACCCTGAAAGTCACCTACGA	108
		R.1338	GAAGGTGATCGGGACGTTGAT	
BC146107.1	*TLN1*	F.942	TTCCTGCCCAAGGAGTATGTG	100
		R.1041	AGCGTACCTTGGCCTCAATCT	
XM_015460928.1	*LGALS8*	F.576	CCCGGTCATCCCCTATGTTG	100
		R.675	CTGGAACCTGTCCGAGTCAC	
XM_003586675.4	*LCN2*	F.393	ACAGTGCGGGTGGTGAACA	100
		R.492	TCCCGTAAAGGGTGATCTTGA	
NM_180998.2	*LTF*	F.631	CCAGGGAGCTGTGGCTAAAT	135
		R.765	AAGTATGGTTCCCGGGAGGA	
NM_001113725.2	*S100A8*	F.19	ATTTTGGGGAGACCTGGTGG	124
		R.142	ACGGCGTGGTAATTCCCTTT	
NM_174197.2	*TLR2*	F.3182	CCATGTCTGGAGAGGGTGTT	102
		R.3283	GGGGACACAAAACAGCACTT	
NM_174814.2	*YWHAZ*	F.	TGAAAATGAAAGGAGACTACTACCG	84
		R.	GCTGTGACTGGTCCACAATC	
NM_173979.3	*ACTB*	F.258	ACCAACTGGGACGACATGGA	149
		R.406	GTCTCGAACATGATCTGGGTCAT	
NM_174178.2	*SDHA*	F.	CTGAAGCAGGTTTCAACACG	113
		R.	GTTGTCCTCCTCCATGTTCC	
NM_174093.1	*IL1B*	F.30	ATTCTCTCCAGCCAACCTTCATT	100
		R.129	TTCTCGTCACTGTAGTAAGCCATCA	
NM_001206735.1	*IL1R*	F.897	CCCGGGCGATAAAGCTGATT	104
		R.1000	AAGCCAGGATCCCAAGACCA	
NM_173966.3	*TNF*	F.174	CCAGAGGGAAGAGCAGTCCC	114
		R.287	TCGGCTACAACGTGGGCTAC	
NM_001034034.2	*GAPDH*	F.908	TTGTCTCCTGCGACTTCAACA	103
		R.1010	TCGTACCAGGAAATGAGCTTGAC	

[1] Primer direction (F – forward; R – reverse) and hybridization position on the sequence are highlighted.

[2] Amplicon size in base pair (bp).

Supplementary Table S2. Sequencing results obtained from PCR product of *Bos taurus* specific primers for genes under investigation.

Gene	Sequence
ITGB2	ATCGAATCGCAGGTGGACAGCCCAGAGGGACTGCGACGGCGTCCAGATCAACGTCCCGATCACCTTCAAGAGGG
TLN1	ACCCCGTCCGCAATTCCTTCTCTACGTGCAGGCACGAGATGCCTAGGAAATGGCTCCCATCCTGTCTCCTCTGCTAAAGGCC
LGALS8	AGCTTGGAACGTTGATTGTACTACGTGGGCATGTTCCTAGTGACTCGGACAGGTTCCAGA
LCN2	CATAGTGTACTTCAAGAAGGTTCAAAGGAAAAAGACGTACTTCAAGATCACCCTTTACGGGAA
LTF	CTCTTTGGCTCTCCCCCGGCCAGAGGGACCTGCTGTTCAAAGACTCTGCTCTTGGGTT
S100A8	GTGTGCCATTAACTCCCTGATTGACGTCTACCACAAGTACTCCCTGAAAAAGGGAATTACCACGCC
TLR2	TTAAAAGAGTCACAATAGAAAGCTTAAGGTTTTTCTGGTTCCTTGTTTCACAACATTTAAATTCGGCGTTAGAATATTTGGATCGTCATAAAAACTTA
ACTB	CACTTCTACACGAGCTGCGTGTGGCCCCCTGAGGAGCACCCCGTGCTGCTGACCGAGGCCCCCTCTGAACCCCAAGGCCAACCGTGAGAAGATGACCCAGATCATGTTCAGAGACA
GAPDH	TCTACCTTCGATGCTGGGGCTGGCATTGCCCTCAACGACCACTTTGTCAAGCTCATTTCCTGGTACGAA
IL1B	ACAGCCATGGCACCGTACCTGAACCCATCAACGAAATGATCGGCTTACGTCACAGTGGACAGAGCACAATAGCACCCCC
IL1R	TGTGAGTCCAGCTAATGAGACAATGGAAGTGGTCTTGGGATCCTGGCTTACAG
TNF	TCACTCTCCGGGGCAGCTCCGGTGGTGGGACTCGTATGCCAATGCCCTCATGGAA

Supplementary Table S3. Average of the amplification efficiencies of the gene used in the study.

Gene	Efficiency
ACTB	1.72
GAPDH	1.82
IL1B	1.78
IL1R	1.87
ITGB2	1.82
LCN2	1.84
LGALS8	1.84
LTF	1.86
S100A8	1.82
SDHA	1.83
TLN1	1.79
TLR2	1.83
TNF	1.77
YWHAZ	1.83

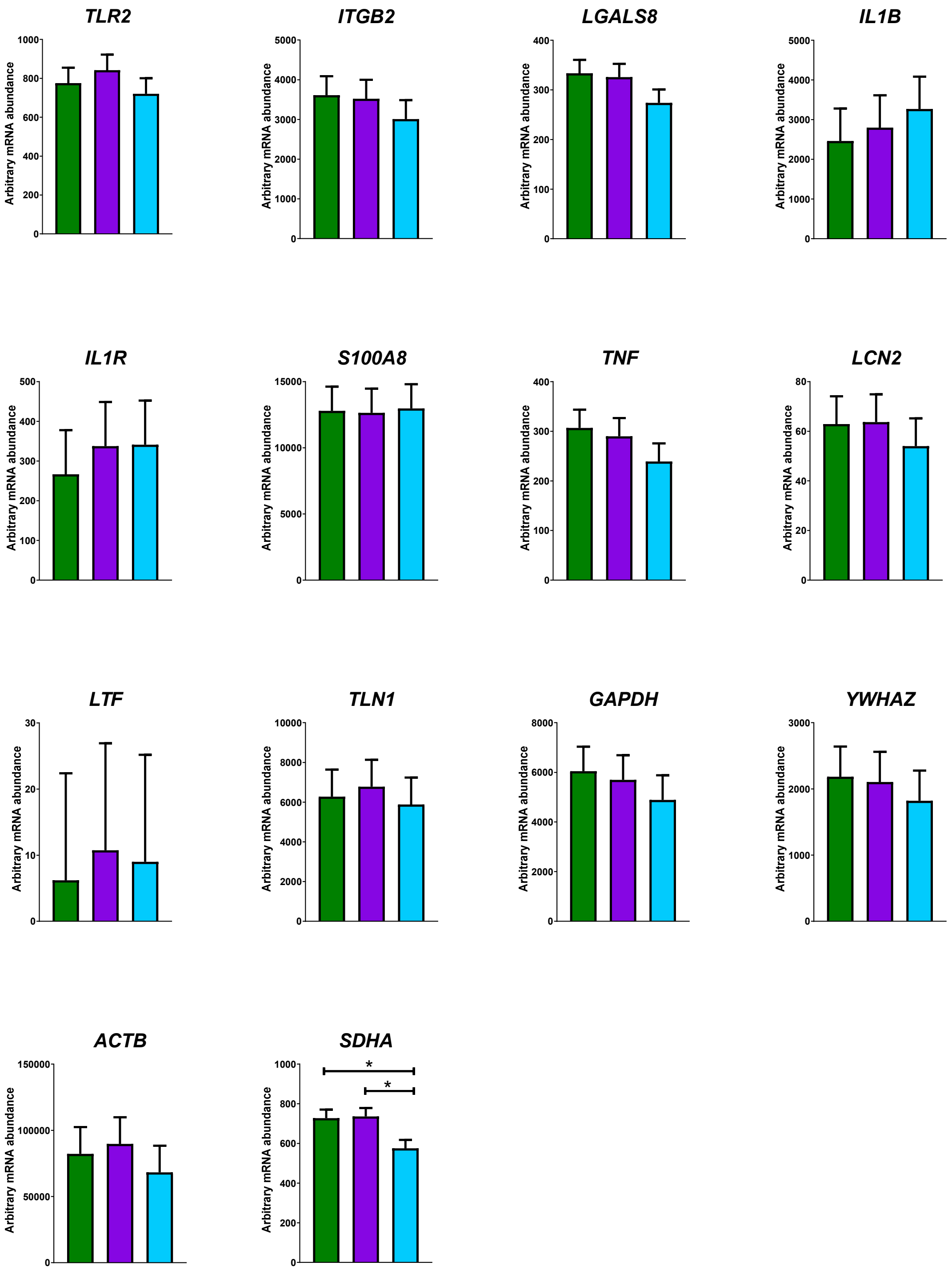

Supplementary Figure S1. Least square means ± SEM of the arbitrary mRNA abundance of genes in leukocytes isolated from blood of mid-lactating Holstein cows collected in Li-heparin (green columns), K-EDTA (purple columns), or Na-citrate (light blue columns) tubes; * indicate significant difference between anticoagulants ($p < 0.05$)
